# Modulation of Malaria Infection in *Anopheles gambiae* Mosquitoes Exposed to Natural Midgut Bacteria

**DOI:** 10.1371/journal.pone.0081663

**Published:** 2013-12-06

**Authors:** Majoline T. Tchioffo, Anne Boissière, Thomas S. Churcher, Luc Abate, Geoffrey Gimonneau, Sandrine E. Nsango, Parfait H. Awono-Ambéné, Richard Christen, Antoine Berry, Isabelle Morlais

**Affiliations:** 1 UMR MIVEGEC (IRD 224- CNRS 5290- UM1- UM2), Institut de Recherche pour le Développement, Montpellier, France; 2 Laboratoire d'entomologie médicale, Organisation de Coordination pour la lutte contre les Endémies en Afrique Centrale, Yaoundé, Cameroon; 3 Department of Infectious Disease Epidemiology, Imperial College London, London, United Kingdom; 4 Université de Douala, Faculté de Médecine et des Sciences Pharmaceutiques, Douala, Cameroon; 5 CNRS UMR 7138, Université de Nice, Faculté des Sciences, Nice, France; 6 Laboratoire de Biologie Virtuelle, UMR 713, Université de Nice, Faculté des Sciences, Nice, France; 7 Service de Parasitologie-Mycologie, Centre Hospitalier Universitaire de Toulouse, Hôpital Rangueil, Toulouse, France; Kansas State University, United States of America

## Abstract

The development of *Plasmodium falciparum* within the *Anopheles gambiae* mosquito relies on complex vector-parasite interactions, however the resident midgut microbiota also plays an important role in mediating parasite infection. In natural conditions, the mosquito microbial flora is diverse, composed of commensal and symbiotic bacteria. We report here the isolation of culturable midgut bacteria from mosquitoes collected in the field in Cameroon and their identification based on the 16S rRNA gene sequencing. We next measured the effect of selected natural bacterial isolates on *Plasmodium falciparum* infection prevalence and intensity over multiple infectious feedings and found that the bacteria significantly reduced the prevalence and intensity of infection. These results contrast with our previous study where the abundance of *Enterobacteriaceae* positively correlated with *P. falciparum* infection (Boissière et al. 2012). The oral infection of bacteria probably led to the disruption of the gut homeostasis and activated immune responses, and this pinpoints the importance of studying microbe-parasite interactions in natural conditions. Our results indicate that the effect of bacterial exposure on *P. falciparum* infection varies with factors from the parasite and the human host and calls for deeper dissection of these parameters for accurate interpretation of bacterial exposure results in laboratory settings.

## Introduction

Malaria remains a major public health burden in developing African countries, with approximately 1 million malaria deaths were reported in 2010 alone [1]. In Sub-saharan Africa, *Plasmodium falciparum* is responsible for the devastating impact of the disease and *Anopheles gambiae* is its main mosquito vector. Malaria parasites are transmitted from human to mosquito when a female *Anopheles* ingests a blood meal from a gametocyte-infected human host. In the mosquito midgut, malaria parasites undergo a series of complex developmental stages and transmission depends on the success of the different transition steps (reviewed in [2], [3]). The major loss of parasites occurs during the ookinete-to-oocyst transition, when the motile ookinetes traverse the midgut epithelium and develop into oocysts resting beneath the basal lamina of the midgut wall [2].

Recently, functional and high-throughput genomic approaches have significantly improved our understanding of vector-parasite interactions that underlie parasite establishment within the mosquito [4]–[9]. Midgut colonization relies on cellular and molecular events but it is now recognized that ecological factors that shape the mosquito distribution play an important role for parasite transmission [10]–[12]. In particular, the mosquito midgut microbiota have been shown to modulate parasite infection [11], [13]–[18]. A protective role of *An. gambiae* midgut bacteria against *Plasmodium* infections was demonstrated when the use of antibiotic treatments to clear the midgut microbiota resulted in enhanced *P. falciparum* infections [5], [19]. By contrast, co-infections of bacteria with *P. falciparum* exhibited a reduced number of developing oocysts in the mosquito midgut, both in laboratory and field studies [5], [13]–[17], [20], [21]. The underlying mechanisms by which bacteria can influence parasite numbers remains elusive. It has been suggested that the interaction between commensal bacteria and the mosquito epithelial cells invaded by *P. falciparum* would elicit immune responses leading to reduced levels of parasite burden, though it could be due to bacteria producing toxins with anti-*Plasmodium* activity [8], [14], [22].

We have previously shown that the composition of the midgut microbiota is a major component that determines mosquito competence, and by contrast to co-infection studies we found in field mosquitoes with native midgut microbiota that the abundance of *Enterobacteriaceae* was positively correlated with the *P. falciparum* infection [11]. Understanding how midgut microbial communities, especially enterobacteria, affect the mosquito vector competence remains a significant challenge.

In the present study, we screened culturable midgut bacteria in field collected *An. gambiae* mosquitoes from Cameroon using the MacConkey agar, which is particularly suitable for the isolation of bacteria from the *Enterobacteriaceae* family. We next examined the role of a set of the identified bacteria strains on *P. falciparum* transmission by feeding bacteria-challenged mosquitoes on blood from naturally-infected gametocyte carriers and provide new findings on the effect of midgut bacteria on malaria parasite development.

## Materials and Methods

### Ethics statement

All procedures involving human subjects used in this study were approved by the Cameroonian national ethical committee (statements 099/CNE/CE/09 and 046/CNE/SE/2012). Children identified as gametocyte carriers were enrolled as volunteers after their parents or legal guardians had signed an informed consent form. All necessary permits were obtained for the described field studies (statement 099/CNE/SE/09 and 046/CNE/SE/2012). Collections did not involve any protected species, and the collecting sites were either public areas or private gardens; in the latter case, the owner gave the permission for collection.

### Collection of mosquitoes and midgut samples

*An. gambiae* mosquitoes were sampled in their aquatic habitats at the L4 and pupae stages. Immature stages were collected in four localities in the vicinity of Yaoundé: Mvan (11°30′28″E, 3°48′22″N, Nkolondom (11°29′49″E, 3°57′20″N), Nkolbisson (11°27′09″E, 3°52′10″N), and Nkolkumu (11°22′18″E, 3°51′02″N). Collections were performed during a two month period, from September to October 2011. Larvae and pupae from each breeding site were kept in a 5-liter container for transportation back to the insectary at the Organisation de Coordination pour la lutte contre les Endémies en Afrique Centrale (OCEAC). Anopheline larvae and pupae were identified morphologically; non anopheline individuals and predators were removed. Pupae were placed in a 20-ml water-containing cup in a 30×30 cm cage for emergence. Larvae were kept in the water from their original habitat in a 3-liter plastic bucket and resulting pupae collected daily for 2 days; adult mosquitoes were collected immediately after emergence.

Larvae, pupae and newly emerged adult (1–2 day old) mosquitoes were selected for midgut microbiota analysis. Mosquitoes were cold-anesthetized and all dissections were processed under sterile conditions. Mosquitoes were surface sterilized with a 70% ethanol solution, as previously reported [23], and then rinsed three times in sterile phosphate-buffered saline (PBS). Midguts were then carefully removed under a stereo microscope (10× magnification) using clean forceps and ground individually in 50 µl of sterile PBS using a single-use pestle.

### Isolation of midgut and environmental bacteria

The MacConkey agar (European Pharm.) was used in this study because of its particular suitability as a selective culture medium for the isolation of Gram-negative bacteria, especially the family of *Enterobacteriaceae*. Each midgut homogenate was plated on MacConkey agar using the streak plate dilution method to obtain isolated bacterial colonies. To retrieve the bacterial content from the water of each breeding site, a 250-ml volume of water collected directly in the field and kept in sterile bottle flask was filtered through Whatman #1 paper. Each filter paper was cut into three pieces and deposited on MacConkey agar, then plated by streaking. All cultures were maintained at 37°C under aerobic conditions for 24 hours. Individual bacterial colonies were re-streaked to ensure single colony isolation. The MacConkey agar medium allows differentiation between lactose-fermenting and non-lactose fermenting Gram-negative bacteria; lactose fermenting bacteria appear as red/pink colonies whereas those non-fermenting bacteria produce white/colorless colonies. Colonies with distinct morphologies, colors and margins were picked and sub-cultured into Luria-Bertani (LB) agar (Invitrogen) to obtain pure bacterial isolates. Single colonies were harvested in LB broth and stored at −80°C as glycerol stocks.

### Genomic DNA extraction, amplification, sequencing and analysis of the 16S rRNA gene

The pure bacterial isolates were sub-cultured once more, 5 colonies per plate were picked and subjected to genomic DNA extraction using the Qiagen DNeasy Blood & Tissue kit (Valencia, CA) according to the manufacturer instructions. Amplification of the 16S rRNA gene from purified genomic DNAs was carried out with the universal primers w18 (5′-GAGTTTGATCMTGGCTCAG-3′) and w02 (5′-GNTACCTTGTTACGACTT-3′) [24]. PCR reactions were performed using the following cycling conditions: an initial denaturation at 94°C for 2 min, 25 cycles at 94°C for 1 min, 50°C for 1 min and 72°C for 1 min, and a final extension at 72°C for 20 min. The expected size of the PCR product, ∼1,400 base pairs (bp), was confirmed by electrophoresis on a 1.5% agarose gel stained with ethidium bromide and visualized by UV transillumination.

Amplicons were purified using the Agencourt AMPure PCR DNA purification kit (Agencourt, Beverly, MA) and subjected to direct bidirectional sequencing with the original primers (w18 and w02). The primers 531F (5′-GTGCCAGCAGCCGCGGT-3′) and 805R (5′-TCGACATCGTTTACGGCGTC-3′) were used as internal primers to obtain high quality full sequences. Sequencing reactions were performed using the BigDye Terminator v3.1 mix and the sequencing products were cleaned with CleanSEQ magnetic beads (Agencourt, Beverly, MA) and analyzed on a 3130xl genetic analyzer (Applied Biosystems, Foster City, CA, USA).

Sequences of the 16S rRNA gene were assembled and analyzed with SeqScape software, version 2.5 (Applied Biosystems). Short read contigs (<700 bp) were removed from the analyses, and potential chimera sequences were screened using Mallard 1.02 Bioinformatics toolkit (available at http://www.softsea.com/download/Mallard.html). Homology searches were perfomed against the Silva reference database (release 111, available at http://www.arb-silva.de/) to identify the closest genetic match for each sequence. Sequences obtained in this study were deposited in GenBank under accession numbers JQ680470 to JQ680962.

### Mosquito maintenance and experimental challenge of mosquitoes with natural bacteria

Mosquitoes of the Ngousso strain of *An. gambiae* were obtained from egg batches that were surface-sterilized by bleaching [25]. All precautions were taken to prevent contaminations by environmental bacteria. Larvae were reared in distilled water and fed with TetraMin Baby food. We checked for resident midgut bacteria by plating midguts from L4 larvae onto chromID CPS (Biomerieux) and LB agar plates. No Gram-positive bacteria were identified, only a few colonies of Gram-negative bacteria corresponding to *Elizabethkingia anophelis*, a bacteria colonizing *An. gambiae* in insectaries [26], were recovered. Adult mosquitoes were maintained on a 6% sucrose solution in sterile PBS at 27±2°C and 85±5% relative humidity with a 12 hours light/dark cycle.

Different bacterial strains isolated from the midgut of wild-caught *An. gambiae* mosquitoes were selected to challenge mosquitoes of the Ngousso strain. We used bacterial strains closely related to *Escherichia*, *Serratia*, *Enterobacter*, *Pseudomonas*, *Acinetobacter* and *Comamonas* genera as they were previously shown to affect pathogen transmission in different insect vectors [14], [17], [23], [27], [28], [29], [30], [31], [32]. Fresh bacterial cultures were prepared from glycerol stocks to challenge mosquitoes. Bacterial isolates were inoculated on LB agar at 37°C for 24 hours, colonies were collected and washed twice in PBS by centrifugation. Optical density (OD) measurements at 600 nm were used to determine bacterial concentration in the culture, with OD600 = 1 representing 10^9^ Colony Forming Units (CFUs)/ml.

### Midgut colonization assays

The *Escherichia coli* isolate JQ680851 was chosen to measure the success of bacterial colonization in mosquito midguts. Two bacterial suspensions, at concentrations of 10^9^ and 10^7^ bacteria/ml were provided on sterile cotton balls to female mosquitoes and the concentration of bacteria in the midgut were determined at different time points post-challenge (at 4, 8, 24, 48, 72 hours). Each female mosquito was surface-sterilized as described above, and the midgut dissected and homogenized in 0.1 ml sterile PBS. The concentration of bacteria was estimated by plating several serial dilutions on ChromID CPS agar plates. After 24 hours incubation in aerobic conditions at 37°C, CFUs per midgut were determined using the equation: (number of colonies count)×(dilution factor)/plated volume (0.1 ml), and expressed as CFUs/ml. Three replicates were performed at different time points for each optical density.

### Experimental infections with natural *P. falciparum* isolates after bacterial challenge

Batches of 2-days-old Ngousso females (80–100 mosquitoes/batch) were fed on cotton balls soaked either with PBS or bacterial cultures (10^9^ bacteria/ml) in 3% sucrose and kept in insectary for 36 hours. Mosquitoes were starved for 18 hours before blood feeding. Bacteria-challenged and PBS-control mosquitoes were fed on gametocyte-containing blood from the same donor to avoid variability in the infection outcomes due to the blood donor [33]. Membrane feedings were performed as previously described, using the AB replacement procedure [11], [34]. Females were allowed to feed for 35 minutes, unfed and partially fed mosquitoes were removed by aspiration and discarded. Engorged females were maintained in the insectary for 8 days, midguts were then dissected, stained with a 0.4% mercurochrome solution and the oocysts enumerated using a light microscope (20× magnification). Infection prevalence was defined as the proportion of mosquitoes harboring at least one oocyst among the total dissected mosquitoes and mean oocyst intensity as the mean number of oocysts per midgut among all dissected mosquitoes.

### Statistical analysis

Statistical analyses were performed using the R statistical software, version 2.15.0 [35]. Hierarchical cluster dendrograms based on Bray-Curtis similarity analysis were performed using the Vegan packages [36]. The bubble plot was constructed using ggplot2 [37] and graphicsQC [38].

We measured the treatment effect on *P. falciparum* infection prevalence and intensity over multiple infectious feedings using generalised linear mixed models (GLMMs). In both the prevalence and intensity models the difference between the PBS control and bacterial challenge was included as a fixed effect whilst overall transmission was allowed to vary at random between hosts. The analysis was repeated for each blood donor separately to see how parasite exposure (as measured by oocyst intensity in the control group) influenced bacterial efficacy. The significance threshold was set at 0.05. A binomial error structure was used to describe changes in oocyst prevalence and a zero inflated negative binomial (ZINB) distribution was used to assess intensity data to accommodate for the over-dispersion in the oocyst counts [39]. Models were fit using the glmmADMB package in R [40].

## Results

### 16S rRNA gene sequences

Culturable bacterial isolates were recovered from 227 individual midguts of mosquitoes collected in four localities and 14 water samples from larval habitats (Table S1). A total of 597 bacterial colonies were isolated from the MacConkey agar plates and 562 (94%) were successfully sub-cultured into LB agar. After sequencing analysis, 464 (84%) high quality sequences of the 16S rRNA gene were obtained, 437 were from midgut samples and 27 from aquatic breeding sites.

The 16S rRNA gene sequences of the bacteria isolated from the mosquito midgut were aligned with published sequences of bacterial strains described in other insect vectors. Sequences from our samples showed high homology to those identified in other insects of medical importance collected in different geographical locations, *An. arabiensis* from Zambia, *An. gambiae* from Kenya, *An. maculipennis* from Iran, *Culex quinquefasciatus* from India, or *Glossina* spp. from Cameroon [13], [14], [23], [29], [31], [41] (Table 1). Interestingly, bacterial strains from certain genera, *Acinetobacter*, *Enterobacter,* showed higher relatedness to strains isolated in *Glossina* spp. from Cameroon [28], [29] than to bacteria strains recovered in other anopheline mosquitoes from more distant areas. For example, the bacterial strain JQ680728 has the best match (99% identity) with *Enterobacter* spp. 11B (HQ289881) from the midgut of *Glossina* spp. collected in Cameroon [29] (Table 1). For other genera, such as *Bacillus*, *Serratia*, and *Pseudomonas*, the 16S rRNA sequences from our isolates showed higher relatedness with sequences of bacteria isolated from *Anopheles* mosquitoes. The *Bacillus* JQ680964 strain has the best match (99% identity) with the JF690927 strain isolated from the midgut of wild-caught *An. arabiensis* from Zambia [14]. The *Serratia marcescens* JQ680856 strain isolated in this study from the midgut of an *An. gambiae* adult female showed 100% similarity with the *Serratia* strain JQ410823, which was isolated from the midgut of *Rhodnius* [42] and 99% similarity with the *Serratia* strain FJ608300, which was isolated from a field-caught *An. stephensi* female from India [32]. The *Pseudomonas* strain JQ680959 that was isolated from pupal midgut was 98% identical to *Pseudomonas mendocina* strain Lma2 isolated from the larvae of *An. maculipennis* from Iran [41]. These results indicate that some bacterial species are closely associated with the insect midguts and the bacteria/insect association may rely either on geographical or taxonomic factors.

**Table 1:** Taxonomic affiliation and abundance of bacterial isolates from midguts of field collected *An. gambiae* or breeding sites.

Phylum	Class	Family	Assigned genus/species^a^	Genbank^b^	Best match^c^	Max id.	Origin of bacterial isolates^d^				
							Male	Female	Pupa	Larva	Habitat
Proteobacteria	β-*proteobacteria*	*Comamonadaceae*	*Delftia sp.*	JQ680815	JF833617.1	100	2	2	0	3	0
		*Comamonadaceae*	*Comamonas sp.*	JQ680958	JF690938.1	96	0	0	1	0	0
	γ*-proteobacteria*	*Moraxellaceae*	*Acinetobacter sp.*	JQ680951	HQ289878.1	99	0	0	3	0	5
			*Acinetobacter septicus*	JQ680956	EF611418.1	99	0	0	1	0	0
		*Aeromonadaceae*	*Aeromonas sp.*	JQ680955	DQ815211.1	100	2	0	2	2	2
			*Aeromonas hydrophila*	JQ680525	GU204971.1	100	0	0	2	14	5
			*Aeromonas veronii*	JQ680933	X74684.1	99	0	0	0	1	3
			*Aeromonas caviae*	JQ680937	HQ407268.1	99	0	0	0	3	2
		*Pseudomonadaceae*	*Pseudomonas stutzeri*	JQ680717	JF431416.1	100	1	2	2	11	0
			*Pseudomonas stutzeri*	JQ680474	AY837753.1	100	1	0	7	31	0
			*Pseudomonas mendocina*	JQ680959	GU204966.1	98	0	0	1	0	0
		*Enterobacteriaceae*	*Enterobacter cloacae subsp.*	JQ680938	EU260136.1	99	0	0	0	0	1
			*Enterobacter sp.*	JQ680728	HQ289881.1	99	1	0	0	1	0
			*Klebsiella sp.*	JQ680797	GQ418030.1	100	2	0	0	0	5
			*Klebsiella sp.*	JQ680795	JF690978.1	94	1	0	0	0	0
			*Salmonella sp.*	JQ680526	EU881982.1	99	0	0	0	0	1
			*Escherichia coli*	JQ680723	JF813185.1	99	2	3	8	15	0
			*Escherichia-Shigella*	JQ680483	GQ867428.1	99	9	6	10	22	0
			*Escherichia-Shigella*	JQ680477	GQ898121.1	99	4	3	14	43	0
			*Escherichia-Shigella*	JQ680851	X80725.1	99	12	13	24	52	3
			*Shigella flexneri*	JQ680484	AB273731.1	99	11	5	7	21	0
			*Rahnella aquatilis*	JQ680494	GU204974.1	99	0	0	0	2	0
			*Serratia marcescens*	JQ680856	JQ410823.1	100	12	11	13	7	0
			*Serratia marcescens*	JQ680863	FJ608300.1	99	2	6	0	0	0
			*Serratia sp.*	JQ680855	JN201947.1	98	0	1	0	0	0

a Affiliation based on the best BLASTn hit. Sequence analyses are based on ∼1.4 kb of the 16S rRNA genes from 464 isolates.

b When multiple 16S rRNA sequences were identical, one was taken as reference sequence.

c Genebank accession number of the best BLASTn hit.

d Number of bacterial isolates from each mosquito stage/gender, habitat is for isolates recovered from the aquatic breeding sites.

### Genetic diversity of *An. gambiae*-associated bacteria in Cameroon

**Figure 1:**
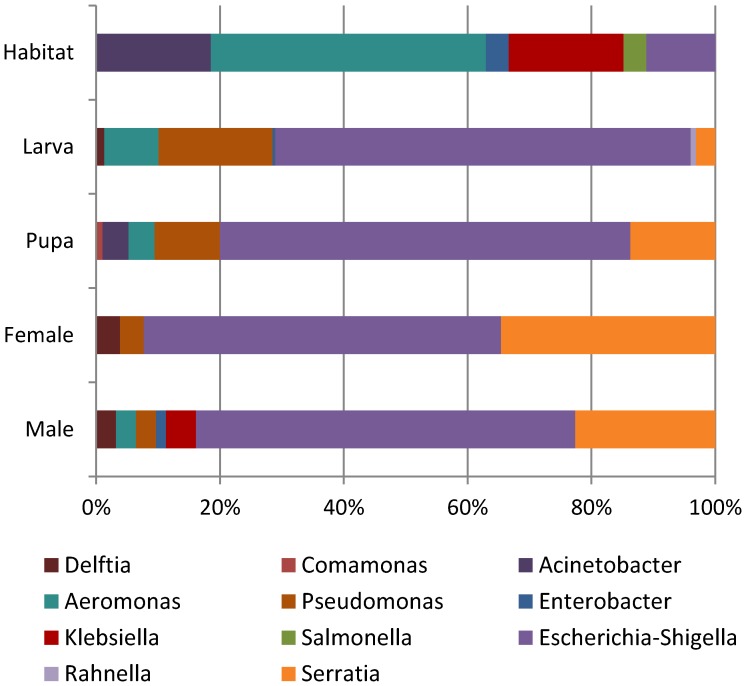
Relative abundance of the bacterial genera within mosquito midguts at each mosquito stage, gender, or within the immature aquatic habitats.

**Figure 2:**
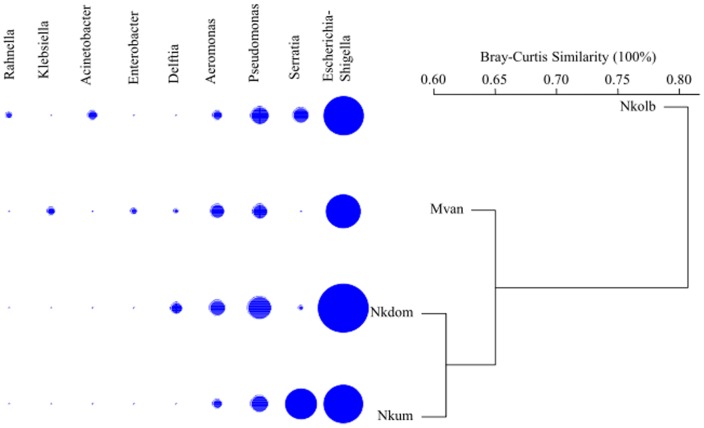
Taxonomic hierarchical classification of the major bacterial groups for each locality. Bray Curtis similarities were calculated for genera abundance and the dendrogram was constructed using the “complete” method. The bubble plot schematically represents major genera abundances (>1%). Nkolb (Nkolbisson), Mvan, Nkdom (Nkolondon), and Nkum (Nkolkumu) indicate the locality where the mosquitoes came from.

The bacterial population of the *An. gambiae* midgut was comprised of members of a single Phylum, Proteobacteria, and specifically the two main classes of *Gammaproteobacteria* (98%) and *Betaproteobacteria* (∼2%). The dominant family identified was the *Enterobacteriaceae* (78%), followed by *Pseudomonadaceae* (12%), *Aeromonadaceae* (6%), *Comamonadaceae* (2%), and *Moraxellaceae* (1%). The current classification of *Escherichia* and *Shigella* genera is based on seven housekeeping genes [43], and we assigned the bacteria to a single group, *Escherichia/Shigella,* when no consensus reference sequence was found upon BLASTn search against the 16 S rRNA sequence database. The main bacterial genera, representing >1% of the sequences, were unevenly distributed among the different mosquito stages or among localities. *Escherichia/Shigella*, *Serratia, Pseudomonas* and *Aeromonas* were over represented. *Escherichia/Shigella*, *Pseudomonas* and *Aeromonas* were particularly abundant in larvae midguts as compared to later stages (Figure 1). Differences in the abundance of bacterial genera identified in the midguts of mosquitoes from the four localities were also observed (Figure 2), with *Serratia* spp. highly represented in mosquitoes from Nkolkumou. Some genera were identified in a single locality: *Enterobacter* and *Klebsiella* in Mvan or *Acinetobacter* and *Rahnella* in Nkolbisson.

In mosquito aquatic habitats, the bacteria were from a single class, *Gammaptoteobacteria*, and belonged to 3 families: *Aeromonadaceae* (44.44%), *Enterobacteriaceae* (37.03%) and *Moraxellaceae* (18.52%) (data not shown). *Aeromonas* were identified in all 4 localities, whereas *Enterobacter* was only found in Mvan, and *Acinetobacter* only in Nkolondom.

### Gut colonization

*In vivo* replication of *Escherichia coli* (strain JQ680851) was assessed after mosquito challenge at different times after exposure. Final *E. coli* concentrations were determined by counting CFUs after plating serial dilutions of midgut homogenates on chromID CPS agar plates. The *E. coli* strain was able to efficiently colonize the mosquito midguts (Figure S1). Bacterial concentrations of *E. coli* decreased for the first 4 hours post-challenge and growth appeared thereafter. *In vivo* replication reached a plateau around 48 hours for the two initial concentrations, indicating a stability of the replication. The number of bacteria per midgut varies between individual mosquitoes, ranging between 10^4^–10^5^ (Dong et al. 2009, Boissiere et al. 2012). Here, the ability of the bacteria to persist in the midgut over time allowed us to test the effect of natural bacterial isolates on *P. falciparum* transmission after colonization of the midgut by bacteria introduced through sugar feeding.

### Effect of bacterial challenge on *P. falciparum* development

The infectiousness of gametocytes depends on multiple parameters such as multiplicity of infection, parasite sex-ratio, parasite density or human factors [34], [44]. We performed multiple replicates assays using different naturally infected gametocyte carriers, to provide an accurate measure of the effect of bacteria on parasite infectivity to mosquitoes. We assayed 7 different midgut bacterial isolates: *Escherichia coli* (JQ680851), *Serratia marcescens* (JQ680856), *Pseudomonas stutzeri* (JQ680796), *Enterobacter* spp. (JQ680715), *Acinetobacter septicus* (JQ680956), *Comamonas* spp. (JQ680958) and *Bacillus pumilus* (JQ680964). *Bacillus pumilus* was isolated from a pupa by plating the midgut on a chromID CPS medium (BioMérieux) in a previous experiment.

**Figure 3:**
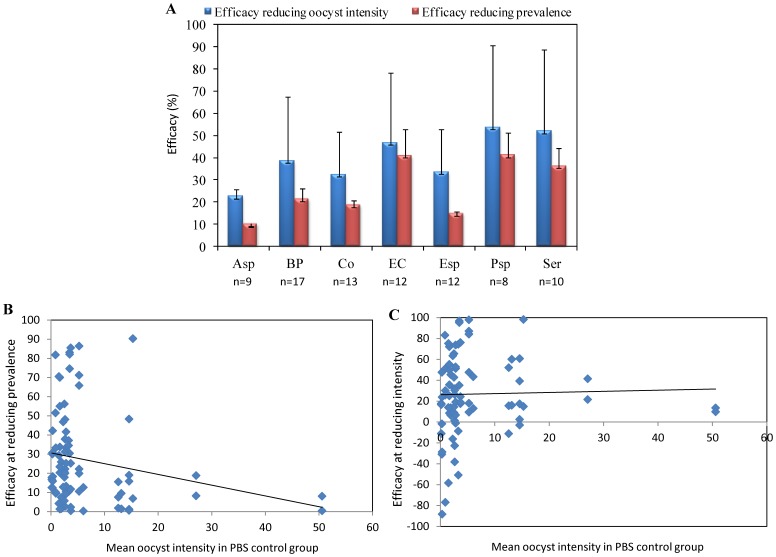
Range of efficacies at reducing oocyst prevalence and intensity. **A** represents the effect of bacterial exposure in the midgut on oocyst intensity and infection prevalence, relative to a PBS control group. The impact of bacterial challenge is shown upon exposure to Asp, *Acinetobacter septicus*; BP, *Bacillus pumilus*; Co, *Comamonas* spp; EC, *Escherichia coli;* Esp, *Enterobacter* spp.; Psp, *Pseudomonas stutzeri*; Ser, *Serratia marcescens.* The number of donors (n) for each treatment is given below the bacterial species. **B** and **C** indicate the correlation between the relative infection prevalence (B) and oocyst intensity (C) and the number of oocysts over all feedings. Each dot indicates the efficacy of a bacteria for a single gametocyte donor.

The effect of bacterial challenge was calculated by comparing oocyst intensity and prevalence in comparison with the PBS control group. Results of the infection prevalence and intensity between bacteria-challenged and control mosquitoes are presented in Tables S2 and S3. Heterogeneity between gametocyte carriers was detected and the combined estimates over all replicates were computed using a mixed-effects model. The effect of exposure to bacteria was significant on both prevalence and intensity of infection for all but one bacterial species. The exposure to *Acinetobacter septicus* did not affect mosquito infection prevalence and only marginally affected oocyst intensity (*P* = 0.011; Table S4). The impact of the bacterial challenge differed between the different bacterial isolates and the impact was more pronounced for *Escherichia coli*, *Serratia marcescens* and *Pseudomonas stutzeri* (Tables S2 and S4, Figure 3A). When efficacy was measured as changes in oocyst intensity, the bacterial challenge had no effect irrespective of the oocyst intensity in the control group (Figure 3B). However, the reduction in oocyst prevalence due to the bacterial exposure was lower when parasite exposure was higher – in feedings that gave rise to the highest oocyst intensities in the PBS control, the bacterial challenge had almost no effect (Figure 3C). The observed difference in parasite inhibition could also be due to different efficiencies in midgut colonization by various bacteria from one feeding to another. However, we used the same mosquito colony and concentrations of bacterial cultures were checked prior each feeding, which renders this possibility unlikely.

## Discussion

Malaria transmission from human host to mosquito vector is complex in natural endemic areas, relying on multiple factors from the mosquito vector, the parasite and the human host [44], [45]. Genetic factors that affect parasite development within the mosquito have been identified [46], [47] but other parameters such as the gametocyte density, the genetic complexity of the parasite isolate or the presence of transmission-reducing antibodies are known to influence the success of the infection [34], [44], [48]. More recently, the role of environmental factors in parasite transmission has gained in interest and, in particular, several studies examined whether the midgut microbiota could modulate *Plasmodium* development in the *Anopheles* mosquito [5], [11], [14]. By using pyrosequencing technology, we have recently described the large diversity of the bacterial communities in the mosquito midgut and we showed from field mosquitoes that the abundance of *Enterobacteriaceae* was higher in *P. falciparum*-infected mosquitoes, indicating that parasites may interact with the resident microbiota [11]. Here, we used culture-dependant methods to characterize the bacterial flora in the midgut of wild mosquitoes and within their breeding sites. We used a selective culture medium, the MacConkey medium, which permits the specific isolation of enteric bacteria. We further investigated the effect of a selected set of midgut bacteria strains on *Plasmodium* transmission by infecting bacteria-challenged mosquitoes with natural isolates of *P. falciparum*.

Microbiota composition is linked to diet and in accordance bacterial communities between males and females were somewhat different. Adult mosquitoes feed on nectar and plant saps and only females require a bloodmeal for egg maturation. The midgut epithelium is covered by a mucus layer that houses the microbiota and previous studies have revealed the important role of commensal bacteria in the vector physiology and immunity [5], [49]. Midgut bacteria play a role in digestive processes for sugar or blood meals [49], [50], and further studies will have to dissect the bacterial flora of wild-caught adult mosquitoes to identify those bacteria that can be acquired from plants in nature. Surprisingly, *Enterobacter* and *Kliebsella* were not identified in female mosquitoes in our study but have already been described as female midgut microbiota-residents [14], [15], [17], [51], [52]. Our findings could indicate that competition between bacterial communities influences the bacterial growth of each species and that the midgut content depends on interactions among species. For feeding, larvae exploit the surface microlayer, a bacteria-rich environment and at molting the midgut epithelium is renewed [53]. Many commensal bacteria from soil environment are then lost during metamorphosis from pupae to adult stages [52]. However, our study and those of others [11], [13]–[15], [17], [18], [31], [32], [41], [51], [52], [54], [55] suggest that the midgut microbiota in immature and adult stages of mosquitoes is diverse and that the gut may not be completely sterilized following adult emergence. In addition, the difference in midgut bacterial composition between field-collected mosquitoes originating from distinct breeding sites indicates that most bacteria are acquired from the environment. Homology searches revealed that the bacteria isolated in the mosquito midgut are more related to strains described in *Glossina* from Cameroon than to others recovered from *Anopheles* mosquitoes from East and West Africa, and this suggests that bacteria can colonize different insect groups within a sympatric geographic area. In contrast to another study that reported a high bacteria diversity in larval habitats using non-culturing methods [52], we found a relative low number of bacteria species in our samples from the mosquito breeding sites and this is likely due to the culture medium used in our study. Deeper investigations of the aquatic habitats are required to determine the mode of acquisition of the mosquito midgut bacteria as different vertical transmission routes have been described in other insects [56], [57].

The resident midgut microbiota play a role in gut homeostasis and changes to the microbial composition can result in damage to the midgut epithelium [8]. In the *Anopheles* vector, bacterial clearance by the use of antibiotics enhances the mosquito susceptibility to *Plasmodium* infection [5], [19]. Interactions between the vector host, the parasite and the microbiota as well as within the microbiota community are not yet understood. The oral infection of mosquitoes with members of the *Enterobacteriaceae* family can render mosquitoes refractory to concomitant *Plasmodium* infection [13]–[15], [17]. In this study, we orally exposed female mosquitoes to bacteria isolated from wild mosquito midguts and the mosquitoes were fed on gametocyte-containing blood from naturally infected human donors 2 days later, when the bacteria had stably colonized the midgut. The exposure to *E. coli*, *Serratia marcescens* and *Pseudomonas stutzeri* strikingly reduced the prevalence and intensity of *P. falciparum* infection. Interestingly, the impact of the bacterial exposure on oocyst prevalence was lower when parasite exposure was higher. We used multiple parasite donors to infect mosquitoes and the different efficiency of bacteria at reducing parasite loads probably results from differences in gametocyte numbers at the time of infection. In a recent report, different strains of *S. marcescens* exhibited variable levels of resistance to *P. berghei* infections [13]. In this study, we provided evidence that the effect of bacterial exposure on *P. falciparum* infection also depends on other factors. The *P. falciparum* isolate-dependent response of the infection could be due to human factors contained in the blood or to different parameters influencing the infectiousness of the gametocytes. Indeed the efficacy of candidate transmission-blocking antibodies decreases with higher parasite intensities and infection intensity varies with the genetic diversity of parasite isolates [34], [39]. Further studies will need to decipher how these different parameters impact *P. falciparum* transmission, and particularly under transmission-blocking antibody pressure, as this would help to predict the effectiveness of control interventions. In this study, we measured the impact of natural midgut bacteria on *P. falciparum* infection for 7 bacterial isolates which represents only a fraction of the bacterial species colonizing the mosquito midgut in the field. We noted that *per os* infection of adult mosquitoes with bacteria probably led to the disruption of gut homeostasis, which, in turn resulted in the failure to control the bacterial bloom and the rapid activation of the innate antimicrobial immune responses in the gut. This limitation points to the importance of considering the natural microbiota and its dynamics when studying microbe-parasite interactions. Malaria transmission in the field involves on an intricate interplay between mosquito, human factors and parasite genotypes, the midgut microbiota and the mosquito innate system. Deciphering the interactions among the different partners and environmental factors in natural conditions will help us develop novel control strategies aimed at blocking *Plasmodium* development within the mosquito vector.

## Supporting Information

Figure S1 ***In vivo*** **replication of** ***Escherichia coli*** **in the midgut of** ***An. gambiae*** **females.** Females, 2-days old, were challenged with bacterial cultures and guts dissected over a period of 72-hours to assess the efficiency of bacterial colonization. Two concentrations of *E. coli* were assayed, 10^9^ and 10^7^ bacteria/ml. Final *E .coli* concentrations were determined by plating serial dilutions of midgut homogenates on chromID CPS agar plates and colony-forming units (CFU) were calculated as described in Materials and Methods. Each point represents the mean ± standard deviation of three replicates.
(TIF)

Table S1 Total number of mosquitoes sampled in each locality at different stages.
(XLS)

Table S2 Mosquito infections in PBS control and bacterial challenged mosquitoes for each gametocyte donor.
(XLS)

Table S3 Mosquito infections in PBS control and bacterial challenged mosquitoes for each gametocyte donor. (suite).
(XLS)

Table S4 Efficacy of bacterial isolates at reducing *Plasmodium falciparum* infection intensity and prevalence.
(XLS)
