# Correction: Modulation of Malaria Infection in *Anopheles gambiae* Mosquitoes Exposed to Natural Midgut Bacteria

**DOI:** 10.1371/annotation/d8908395-a526-428c-b9ed-4430aaf8f7d7

**Published:** 2013-12-30

**Authors:** Majoline T. Tchioffo, Anne Boissière, Thomas S. Churcher, Luc Abate, Geoffrey Gimonneau, Sandrine E. Nsango, Parfait H. Awono-Ambéné, Richard Christen, Antoine Berry, Isabelle Morlais

There were multiple errors in the second paragraph of the Effect of bacterial challenge on *P. falciparum* development section of the Results: 

"When efficacy was measured as changes in oocyst intensity, the bacterial challenge had no effect irrespective of the oocyst intensity in the control group (Figure 3C). However, the reduction in oocyst prevalence due to the bacterial exposure was lower when parasite exposure was higher – in feedings that gave rise to the highest oocyst intensities in the PBS control, the bacterial challenge had almost no effect (Figure 3B)."

In addition, an affiliation of the ninth author is missing: In addition to institution number 7, Antoine Berry is also affiliated with the the following institution: Centre de Physiopathologie de Toulouse-Purpan, Institut National de la Santé et de la Recherche Médicale, UMR1043, CNRS UMR5282, Université de Toulouse Paul Sabatier, Toulouse, France.

