# Supplementary figures and images for: Modulation of Malaria Infection in Anopheles gambiae Mosquitoes Exposed to Natural Midgut Bacteria
Source: PLoS One. 2013 Dec 6;8(12):e81663. doi: 10.1371/journal.pone.0081663 (PMC3855763; doi:10.1371/journal.pone.0081663)

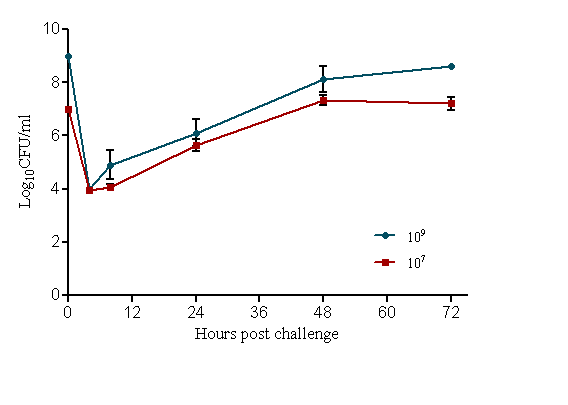

Supplement: Figure S1 — In vivo replication of Escherichia coli in the midgut of An. gambiae females. Females, 2-days old, were challenged with bacterial cultures and guts dissected over a period of 72-hours to assess the efficiency of bacterial colonization. Two concentrations of E. coli were assayed, 109 and 107 bacteria/ml. Final E .coli concentrations were determined by plating serial dilutions of midgut homogenates on chromID CPS agar plates and colony-forming units (CFU) were calculated as described in Materials and Methods. Each point represents the mean ± standard deviation of three replicates. (TIF) [file pone.0081663.s001.tif]
